# Heat stress impacts the multi-domain ruminal microbiota and some of the functional features independent of its effect on feed intake in lactating dairy cows

**DOI:** 10.1186/s40104-022-00717-z

**Published:** 2022-06-15

**Authors:** Tansol Park, Lu Ma, Shengtao Gao, Dengpan Bu, Zhongtang Yu

**Affiliations:** 1 Department of Animal Sciences, The Ohio State University, Columbus, OH USA; 2 Department of Animal Science and Technology, Chung-Ang University, Anseong-si, Gyeonggi-do Republic of Korea; 3 State Key Laboratory of Animal Nutrition, Institute of Animal Science, Chinese Academy of Agricultural Sciences, Beijing, 100193 People’s Republic of China; 4 CAAS-ICRAF Joint Lab on Agroforestry and Sustainable Animal Husbandry, Beijing, 100193 People’s Republic of China

**Keywords:** Functional profiles, Heat stress, Microbiome, Multi-kingdom, Network analysis, Ruminal microbiota

## Abstract

**Background:**

Heat stress (HS) affects the ruminal microbiota and decreases the lactation performance of dairy cows. Because HS decreases feed intake, the results of previous studies were confounded by the effect of HS on feed intake. This study examined the direct effect of HS on the ruminal microbiota using lactating Holstein cows that were pair-fed and housed in environmental chambers in a 2 × 2 crossover design. The cows were pair-fed the same amount of identical total mixed ration to eliminate the effect of feed or feed intake. The composition and structure of the microbiota of prokaryotes, fungi, and protozoa were analyzed using metataxonomics and compared between two thermal conditions: pair-fed thermoneutrality (PFTN, thermal humidity index: 65.5) and HS (87.2 for daytime and 81.8 for nighttime).

**Results:**

The HS conditions altered the structure of the prokaryotic microbiota and the protozoal microbiota, but not the fungal microbiota. Heat stress significantly increased the relative abundance of Bacteroidetes (primarily Gram-negative bacteria) while decreasing that of Firmicutes (primarily Gram-positive bacteria) and the Firmicutes-to-Bacteroidetes ratio. Some genera were exclusively found in the heat-stressed cows and thermal control cows. Some co-occurrence and mutual exclusion between some genera were also found exclusively for each thermal condition. Heat stress did not significantly affect the overall functional features predicted using the 16S rRNA gene sequences and ITS1 sequences, but some enzyme-coding genes altered their relative abundance in response to HS.

**Conclusions:**

Overall, HS affected the prokaryotes, fungi, and protozoa of the ruminal microbiota in lactating Holstein cows to a different extent, but the effect on the structure of ruminal microbiota and functional profiles was limited when not confounded by the effect on feed intake. However, some genera and co-occurrence were exclusively found in the rumen of heat-stressed cows. These effects should be attributed to the direct effect of heat stress on the host metabolism, physiology, and behavior. Some of the “heat-stress resistant” microbes may be useful as potential probiotics for cows under heat stress.

**Supplementary Information:**

The online version contains supplementary material available at 10.1186/s40104-022-00717-z.

## Background

Global warming as a result of the increased release of greenhouse gases (GHG) from different sources, including agriculture and livestock, is of great concern worldwide. Animal production, including dairy production, is impaired considerably by rising environmental temperature. The elevated ambient temperature accompanying global warming has been increasing the frequency and duration of heat stress (HS) episodes in dairy cows, especially in tropical, subtropical, and Mediterranean regions [1]. Dairy cows are more susceptible to HS than other farm animals, and they suffer from HS when the average temperature-humidity index (THI) exceeds 68 (approximately 22 °C at 50% relative humidity) [2]. Heat stress can have profound adverse effects on many aspects of dairy cows, including decreasing feed intake, feed efficiency, nutrient digestibility, milk production, milk quality; creating negative energy balance; and impairing reproductive performance among others [3–6]. Any of these adverse effects can lead to significant economic loss to the producers [7]. Moreover, HS also leads to poor animal welfare [8]. Heat stress in dairy cows is worsening as global warming continues, and HS is now well recognized as an environmental stressor that undermines the sustainable development of the dairy industry [9]. Therefore, HS in dairy cows has attracted tremendous research interest in the past decade. It has been shown that HS can directly impair the normal metabolism, physiology, and immune system in lactating dairy cows, including the metabolism of lipid in bovine primary adipocytes [10], the metabolism of carbohydrate and lipid, and milk protein synthesis in mammary tissues [11, 12], the metabolism of glucose and energy and energy balance [13], and immune system function [14]. Reduced voluntary feed intake is inherently associated with HS, and it aggravates these adverse effects indirectly [15–17].

The complex and diverse ruminal microbiota [18–20] plays a pivotal role in supplying the majority of the energy and nutrients required by dairy cows [21]. This microbiota is also dynamic responding to many internal and external factors [22–24], including HS [25, 26]. Heat stress can affect the ruminal microbiota because it reduces feed intake and impacts the metabolism, physiology, immune system, and behavior of the host. Using 16S rRNA or its gene-based analysis, the earliest studies showed that HS significantly affected the species richness and composition of the ruminal microbiota in dairy heifers with a BW ≥ 430 kg [27] and increased the genus *Streptococcus* and *Clostridium coccoides*–*Eubacterium rectale* group but decreased *Fibrobacter* in the rumen of dairy heifers [28]. In recent years, metataxonomics and metagenomics have been used to comprehensively examine how the ruminal microbiota responds to HS. Zhao et al. [25] found that HS did not significantly alter the bacterial community structure but affected some bacteria, including lactate-producing bacteria (increase) and acetate-producing bacteria (decrease) in the rumen of lactating dairy cows. Another recent study showed that increasing THI did not significantly affect the α-diversity of the ruminal microbiota in goats but shifted the population of some rumen bacteria, including an increase of Bacteroidetes at the expense of Firmicutes [29]. This study also showed that elevated THI could affect the function of the ruminal microbiota as predicted using PICRUSt. Baek et al. [30] evaluated the effect of HS on the ruminal microbiota of Hanwoo steers, and they showed delayed ruminal microbiota response: significant effects after six days but not after three days. In a comparative study [26], HS was shown to affect the ruminal microbiota and its predicted function in lactating Holstein cows and Jersey cows to a different extent. The reported studies provided useful insights into the effects of HS on the ruminal microbiota, but the responses of the ruminal microbiota to HS varied considerably.

Many factors, such as THI, feed and feeding, species or breed of animals, can affect the responses of the ruminal microbiota to HS. In particular, HS decreases feed intake substantially [16, 17], and feed intake can profoundly affect the ruminal microbiota [31–33]. To the best of our knowledge, the animals were fed ad libitum in all the published studies that examined the effect of HS on the ruminal microbiota. Therefore, the decreased feed intake associated with heat-stressed animals but not control animals is a significant confounding factor that prevents the appraisal of the direct HS effect on the ruminal microbiota. We hypothesized that the actual effect of HS on the ruminal microbiota could only be determined when cows were fed the same diet and at the same feed intake. We also hypothesized that HS could also affect rumen methanogens, protozoa, and fungi differently, none of which has been targetedly analyzed in heat-stressed ruminant animals. In the present study, we tested the above hypotheses using lactating cows that were pair-fed the same diet using metataxonomics targeting all the kingdoms of the ruminal microbiota. Our results provided the first insights into how HS could affect the ruminal microbiota independent of the indirect effect on feed intake.

## Methods

### Animals and heat stress treatments

The animal trial of heat stress has been reported previously [11]. Briefly, animals were cared for per the guidelines of the institutional animal care and use committee of the Institute of Animal Science, Chinese Academy of Agricultural Sciences. Four multiparous, lactating Holstein cows (101 ± 10 d in milk, 574 ± 36 kg of body weight, 38 ± 2 kg of milk/d, second parity, 1–2 month pregnant) were individually housed in four environmental chambers (temperature fluctuation: ± 0.5 °C, relative humidity fluctuation: ± 5%). The chambers had 12 h light:12 h dark cycles. The cows were initially fed twice daily (0500 and 1700 h) a total mixed ration (TMR: hay, 24.0%; whole corn silage, 26.0%; steam-flaked corn, 22.0%; extruded soybean, 2.1%; bean pulp, 11.4%; rapeseed and corn meal, 12.4%; limestone and salt, 1.2%; salt, 0.38%; and supplement 0.6%) ad libitum and allowed to adapt to the chamber (maintained at thermal neutral conditions: 20 °C, 40% humidity) for 9 d. Then the four cows were randomly allocated to 1 of 2 treatments in a 2 × 2 crossover design: PFTN (20 °C, 55% humidity; THI = 65.5) and cyclic HS conditions (55% humidity; 06:00–18:00 h at 36 °C while 18:00–06:00 h at 32 °C; THI = 87.2 and 81.8, respectively). THI was calculated as THI = (0.8 × air temperature in °C) + relative humidity × (air temperature °C – 14.3) + 46.4 [34, 35]. All the cows were fed the same TMR formulated to meet or exceed the predicted requirements [36] of energy, protein, minerals, and vitamins. The animals in the HS group were fed ad libitum, while those in the PFTN group were fed the amount based on the intake of the HS group of the previous day. During the experiment, all the animals had free access to drinking water, and they were milked twice daily (05:00 and 17:00 h). Each of the two experimental periods lasted for 18 d, and a 30-d wash-out period (thermoneutral conditions at 20 °C and 55% humidity, THI = 65.5, with ad libitum feeding) separated the two experimental periods. Rumen fluid samples were collected from individual cows three hours after the morning feeding on d 9 of each treatment period using stomach tubing (the first 50 mL of rumen fluid was discarded to avoid saliva contamination) [37]. The rumen samples were immediately stored at − 80 °C until metagenomic DNA extraction.

### Metagenomic DNA extraction and metataxonomic analysis of the ruminal microbiota

Metagenomic DNA was extracted from each rumen sample using the RBB + C method [38]. The quality and quantity of the extracted DNA were determined using a NanoDrop ND-2000 spectrophotometer (Thermo Scientific, NanoDrop Technologies, Wilmington, DE, USA) followed by agarose gel (1%, w/v) electrophoresis. The rumen prokaryotic, protozoal, and fungal microbiotas were analyzed using metataxonomics and separate amplicon libraries prepared using PCR primers specific for the V4 region of 16S rRNA genes of bacteria and archaea, ITS1 of fungi, and the V3-V4 region of the 18S rRNA gene of protozoa, respectively [22]. The primer information and respective PCR conditions were presented in our previous study [22]. The amplicon libraries containing unique barcodes for multiplexing were pooled and sequenced using the 2 × 300 paired-end protocol on an Illumina MiSeq platform at the Molecular and Cellular Imaging Center of The Ohio State University following the Illumina protocol [39].

The sequence reads were analyzed using QIIME2 (version 2019.4) [40] as done in a previous study [22]. Briefly, after trimming off the adapter and primer sequences using Cutadapt [41], DADA2 was used to perform quality filtering (Q-score > 25), merging of paired-end reads, and removal of chimeric sequences [42]. For the fungal ITS1 sequence reads, ITSxpress [43] was used to trim the ITS1 region from paired-end reads resulting in a single merged FASTQ file followed by using the q2-dada2 denoise-single method [42] for further dereplication and chimeric sequence removal. For prokaryotic sequences, further taxonomic filtration was done to remove the amplicon sequence variants (ASVs) labeled as ‘unassigned’, ‘chloroplast’, or ‘mitochondria’. The resulting prokaryotic and fungal ASVs from each denoised multi-kingdom library were taxonomically assigned using respective taxonomic classifiers that had been manually trained using the Naïve Bayes classifier [44] with the Greengenes 16S reference database (13_8 version) [45] for bacteria and archaea, UNITE ITS reference sequences (v.8.0 11.18.2018 database) [46] for fungi. Protozoal ASVs were classified using BLASTn against the NCBI nucleotide collections excluding uncultured/environmental sample sequences (accessed March 6, 2021), and the best BLASTn hits were selected for assigning the taxonomy of each protozoal ASV. Prevalent phyla and genera found in at least 50% of the rumen samples were mainly discussed in this study.

α-Diversity metrics including species richness (Chao1 estimates, observed ASVs, observed genera, and observed species), evenness, Faith’s phylogenetic diversity, Shannon’s index, and Simpson’s index were calculated based on the rarefied ASV BIOM tables. Comparison of the overall microbiotas between the PFTN control cows and the HS cows were done using principal coordinates analysis (PCoA) based on weighted UniFrac distances [47]. The number of shared genera between the two treatments and exclusively found in each treatment were visualized using a Venn diagram. Multi-kingdom co-occurrence and mutual-exclusion relationships were analyzed based on prevalent microbial genera (present in at least 50% of the rumen samples) using FastSpar by computing the correlation with the SparCC algorithm for compositional data (https://github.com/scwatts/fastspar) [48]. Of the microbial interactions with tendency (*P* ≤ 0.1), the microbial networks specific to each treatment were defined and visualized using co-expression differential network analysis (CoDiNA) [49].

### Prediction of functional profiles of the prokaryotic and fungal microbiotas

Microbial functional profiles were predicted from 16S- and ITS1-based ASVs using PICRUSt2 [50] using the reference genomes of the Integrated Microbial Genomes (IMG) database implemented in PICRUSt2. The normalized counts of predicted Enzyme Commission (EC) numbers for both prokaryotic and fungal microbiotas were used to define the overall functional dissimilarities between the PFTN control cows and the HS cows and then analyzed using principal components analysis (PCA) based on the Bray-Curtis dissimilarity metrics [51]. The PCA results were visualized as plots using the ggfortify package of R (3.5.3) [52].

### Statistical analysis

α-Diversity metrics of each kingdom microbiota (prokaryotic, fungal, or protozoal), Firmicutes-to-Bacteroidetes ratio, and the number of PICRUSt2-predicted functional features were statistically analyzed with treatment (i.e., thermal condition), period, and treatment × period interaction as fixed effects and cow as random effect using the GLIMMIX procedure of SAS 9.4 (SAS Institute Inc., Cary, NC, USA). The comparative analysis results of PCoA between the two thermal conditions were further assessed using permutational multivariate analysis of variance (PERMANOVA) with 999 random permutations. Differentially abundant microbial phyla, genera, and EC numbers between the PFTN control cows and HS cows were defined using linear discriminant analysis effect size (LefSe) [53] with logarithmic LDA score 2 as the cutoff. Statistical significance was declared at *P* ≤ 0.05 and tendency at 0.05 < *P* ≤ 0.10.

## Results

As presented in our previous study [11], the HS conditions significantly increased respiratory rates (33.3 bpm for FFTN vs. 77.8 bpm for HS, *P* < 0.0001) and rectal temperature (38.5 °C for FFTN vs. 40.0 °C for HS, *P* = 0.0026). By using the PFTN group as control to eliminate the confounding difference in DMI, comparison between PFTN and HS allowed for the revelation of the true effect of heat stress on the multi-kingdom rumen microbiome.

After denoising and quality filtration, on average > 12,000 sequences were each obtained for prokaryotes and fungi, and > 21,000 sequences were retained for protozoa for each sample (Additional file 1: Table S1). Based on the fewest numbers of sequences for each kingdom among all the samples, 8941, 10,691, and 12,120 sequences per sample were rarefied and analyzed for prokaryotes, fungi, and protozoa, respectively. Good’s coverage for kingdoms and all the samples reached over 99.9% (data not shown).

### Heat stress affects the diversity and co-occurrence interactions of the ruminal microbiota

Heat stress did not affect any of the α-diversity metrics for prokaryotic or fungal microbiota, but it tended to lower the evenness (*P* = 0.057) and Shannon’s index (*P* = 0.075) of the protozoal microbiota compared to the pair-fed thermal neutral (PFTN) control cows, while more protozoal genera (*P* = 0.004) were observed in HS (Table 1). Period had some minor effect on the evenness of the prokaryotic microbiota (*P* = 0.045), observed species richness (*P* = 0.085), and Faith’s phylogenetic diversity (*P* = 0.084) of fungi. The evenness (*P* = 0.001), Shannon’s index (*P* = 0.001), and Simpson’s index (*P* = 0.009) of protozoal microbiota were all affected by period (Table 1).

**Table 1:** α-Diversity metrics of ruminal multi-kingdom microbiota in heat-stressed (HS) cows and pair-fed thermoneutral (PFTN) control cows

Measurements	Treatments (Trt^a^)		SEM	*P*-values		
	PFTN	HS		Trt	Period (P)	Trt × P
Bacteria and archaea						
Chao1 estimates	259	249	14.57	0.706	0.437	0.061
Observed ASVs	259	248	14.42	0.704	0.426	0.060
Observed genera	62	55	2.78	0.210	0.181	0.342
Observed species	66	59	3.02	0.222	0.194	0.370
Evenness	0.87	0.87	0.01	0.927	**0.045**	0.935
Faith’s phylogenetic diversity	21.57	19.92	0.98	0.365	0.274	0.204
Shannon’s index	6.95	6.88	0.11	0.724	0.124	0.406
Simpson’s index	0.98	0.98	0	0.992	0.105	0.770
Fungi						
Chao1 estimates	81	83	5.31	0.837	0.155	0.327
Observed ASVs	81	83	5.32	0.888	0.136	0.358
Observed genera	44	43	3.82	0.897	0.105	0.584
Observed species	50	50	4.66	0.979	0.085	0.592
Evenness	0.69	0.73	0.02	0.222	0.414	0.164
Faith’s phylogenetic diversity	20.05	20.10	1.48	0.987	0.084	0.418
Shannon’s index	4.31	4.67	0.14	0.184	0.123	0.245
Simpson’s index	0.89	0.92	0.01	0.100	0.900	0.145
Protozoa						
Chao1 estimates	19	19	0.874	0.819	0.631	0.806
Observed ASVs	19	19	0.861	0.793	0.602	0.746
Observed genera	5.5^b^	6.13^a^	0.164	**0.004**	0.473	0.089
Observed species	10	10	0.272	0.633	0.074	0.347
Evenness	0.66	0.53	0.044	0.057	**0.001**	0.574
Faith’s phylogenetic diversity	1.56	1.42	0.172	0.732	0.976	0.630
Shannon’s index	2.76	2.27	0.187	0.075	**0.001**	0.483
Simpson’s index	0.76	0.65	0.047	0.134	**0.009**	0.377

^*^ Thermal treatment (heat stress vs. pair-fed thermoneutrality)

Good’s coverage of all samples was ≥99.9%

Mean values with different superscript in a row differ (*P* < 0.05)

**Fig. 1:**
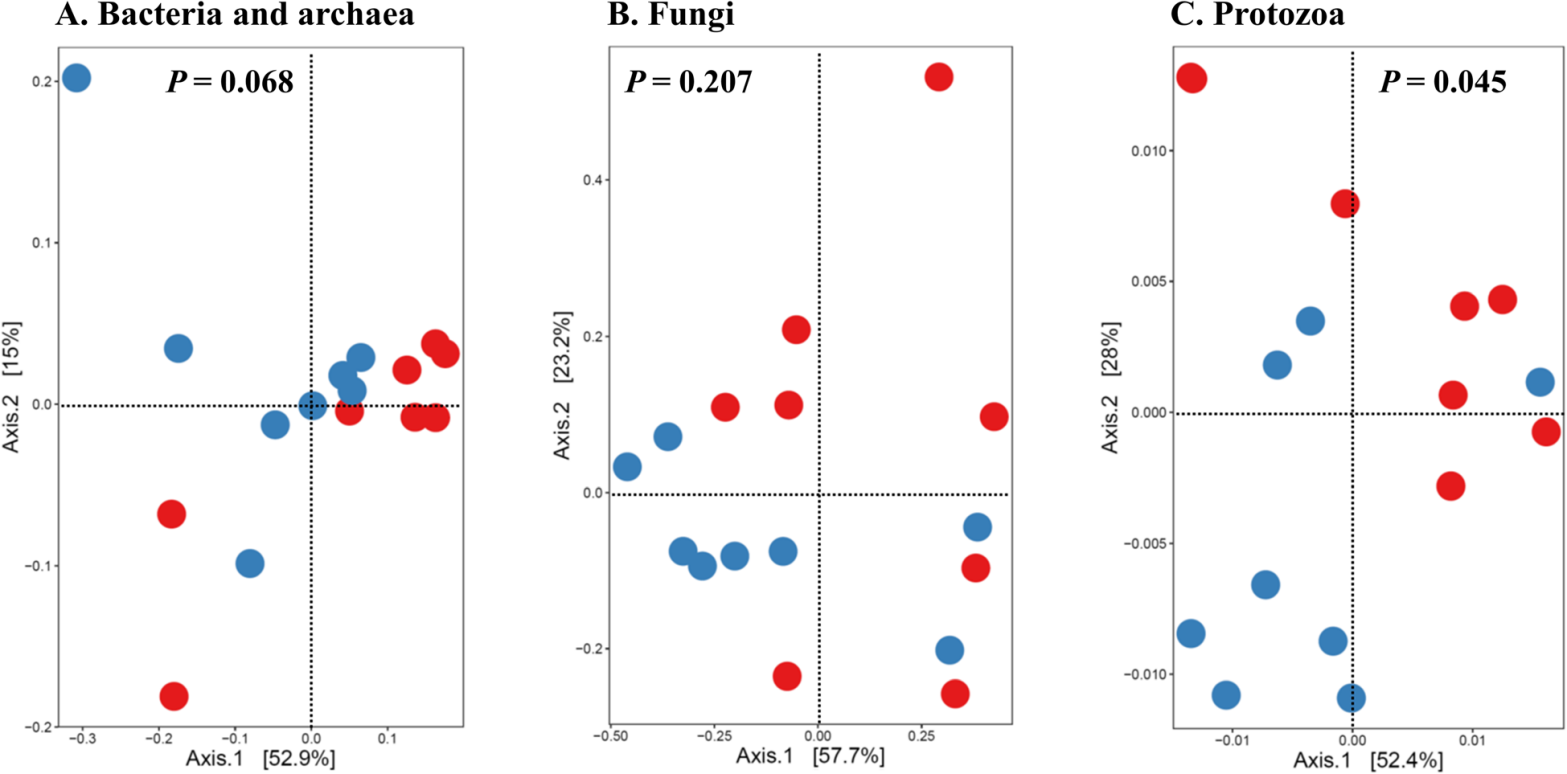
Principal coordinates analysis (PCoA) plots (based on weighted UniFrac distance metrics) showing the comparison of the ruminal microbiota of bacteria and archaea (**A**), fungi (**B**), and protozoa (**C**) between the heat-stressed cows (red dot) and the pair-fed thermal neutral control cows (blue dot). The *P*-values were based on PERMANOVA analysis

**Fig. 2:**
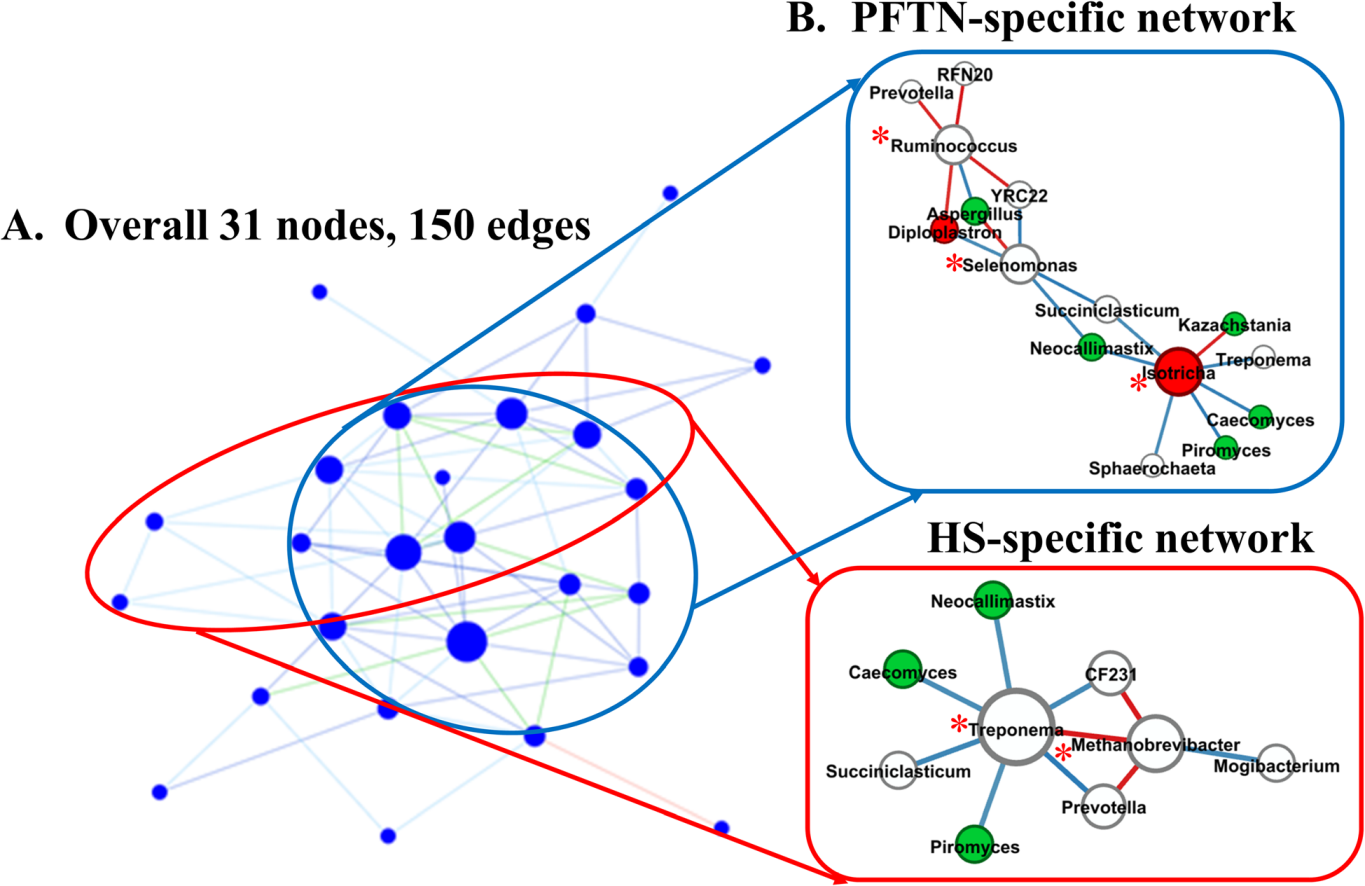
Co-occurrence and mutual exclusion network based on significant correlations (*P* ≤ 0.1) of relative abundance of major classified genera (each detected in at least 50% of the sample) of ruminal microbiota of pair-fed thermal neutral (PFTN) control cows and heat-stressed (HS) cows (**A**), and the genus-level nodes exclusively found in the PFTN cows and the HS cows (**B**). The size of the nodes represents the sum of the weights of the interactions connected. The exclusive nodes were marked as red asterisks within each exclusive microbial network. Blue and red lines indicate co-occurrence and mutual exclusion, respectively

Based on PCoA and PERMANOVA analyses, compared to PFTN, HS altered the overall prokaryotic microbiota with a trend toward statistical significance (*P* = 0.068) and the protozoal microbiota significantly (*P* = 0.045), but it did not affect (*P* = 0.207) the overall fungal microbiota (Fig. 1). Co-occurrence network analysis using CoDiNA identified 17 and 10 significant interactions among 150 overall edges exclusively found in the ruminal microbiotas of the PFTN control and the HS cows, respectively (Fig. 2A). Three genus-level nodes were found only in the PFTN control cows; they included two bacterial genera: *Ruminococcus* and *Selenomonas,* and one protozoal genus *Isotricha*. Only two genus-level nodes were exclusively found in the HS cows: one archaeal genus *Methanobrevibacter* and one bacterial genus *Treponema*. No fungal nodes were exclusively found in either the PFTN control cows or the HS cows. Of the three genus nodes specific for the PFTN control cows (Fig. 2B), *Ruminococcus* was mutually exclusive with three bacterial genera (i.e., *Prevotella*, YRC22, and RFN20) and one protozoal genus (i.e., *Diploplastron*), but it showed co-occurrence with *Aspergillus*. On the other hand, *Selenomonas* was mutually exclusive with *Aspergillus*, and it had co-occurrence with four genera: YRC22, *Succiniclasticum*, *Neocallimastix*, and *Diploplastron*. The only specific genus node, *Isotricha*, had seven significant interactions, including co-occurrence with three genera of bacteria (i.e., *Succiniclasticum*, *Sphaerochaeta*, and *Treponema*) and three genera of fungi (i.e., *Neocallimastix*, *Piromyces*, and *Caecomyces*), and mutual exclusion with *Kazachstania* (a genus of yeast). In the specific network of HS cows (Fig. 2B), the exclusive node *Treponema* showed co-occurrence with three rumen fungal genera (*Caecomyces*, *Neocallimastix*, and *Piromyces*) and three bacterial genera (*Prevotella*, CF231, and *Succiniclasticum*). Another exclusive node of the network of HS cows, *Methanobrevibacter*, was mutually exclusive with two Bacteroidetes genera (*Prevotella* and CF231) while co-occurring with *Mogibacterium*. Those two exclusive nodes (*Treponema* and *Methanobrevibacter*) were mutually exclusive.

### Heat stress affects some of the taxa of the ruminal microbiota

**Fig. 3:**
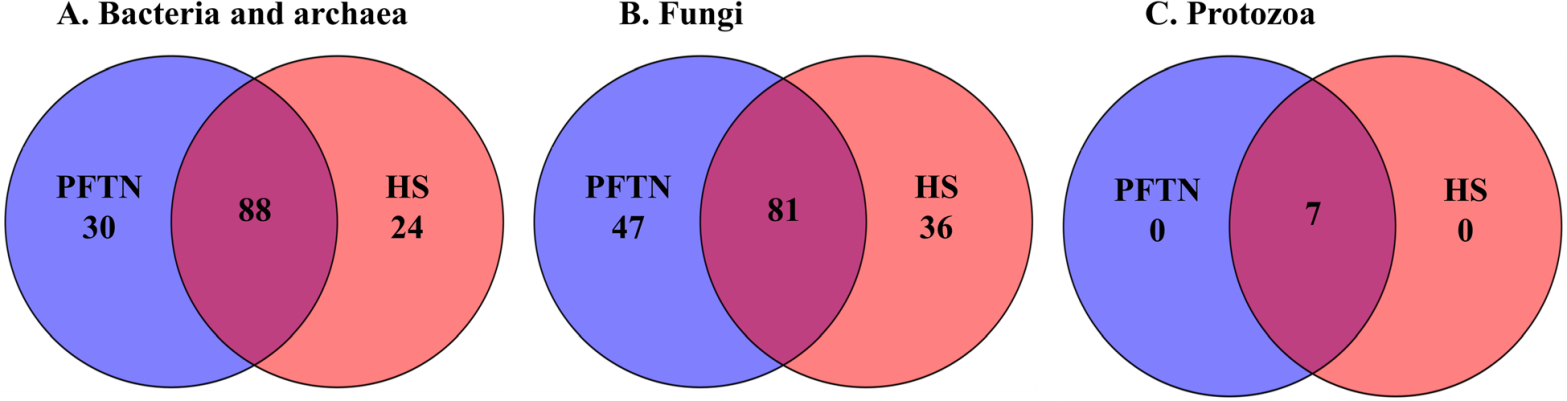
Venn diagrams showing the genera of rumen microbes shared between and unique to the heat-stressed (HS) cows and the pair-fed thermal neutral (PFTN) control cows

Comparison using Venn diagrams revealed the microbial genera (both taxonomically classified and unclassified genus-level equivalents) shared by or exclusively detected in the PFTN control cows and the HS cows (Fig. 3). In the prokaryotic microbiota, 88 of the 142 detected genera were shared between the two thermal conditions, and so were all the three identified archaeal genera. Thermoneutrality and HS corresponded to 30 and 24 bacterial genera that were exclusively found therefor. Of the 164 detected fungal genera (including taxonomically unclassified genus-level equivalents), 81 were shared, whereas 47 and 36 were exclusively found in the PFTN control cows and the HS cows, respectively. Only seven protozoal genera were detected, and all of them were shared between the two thermal conditions.

**Fig. 4:**
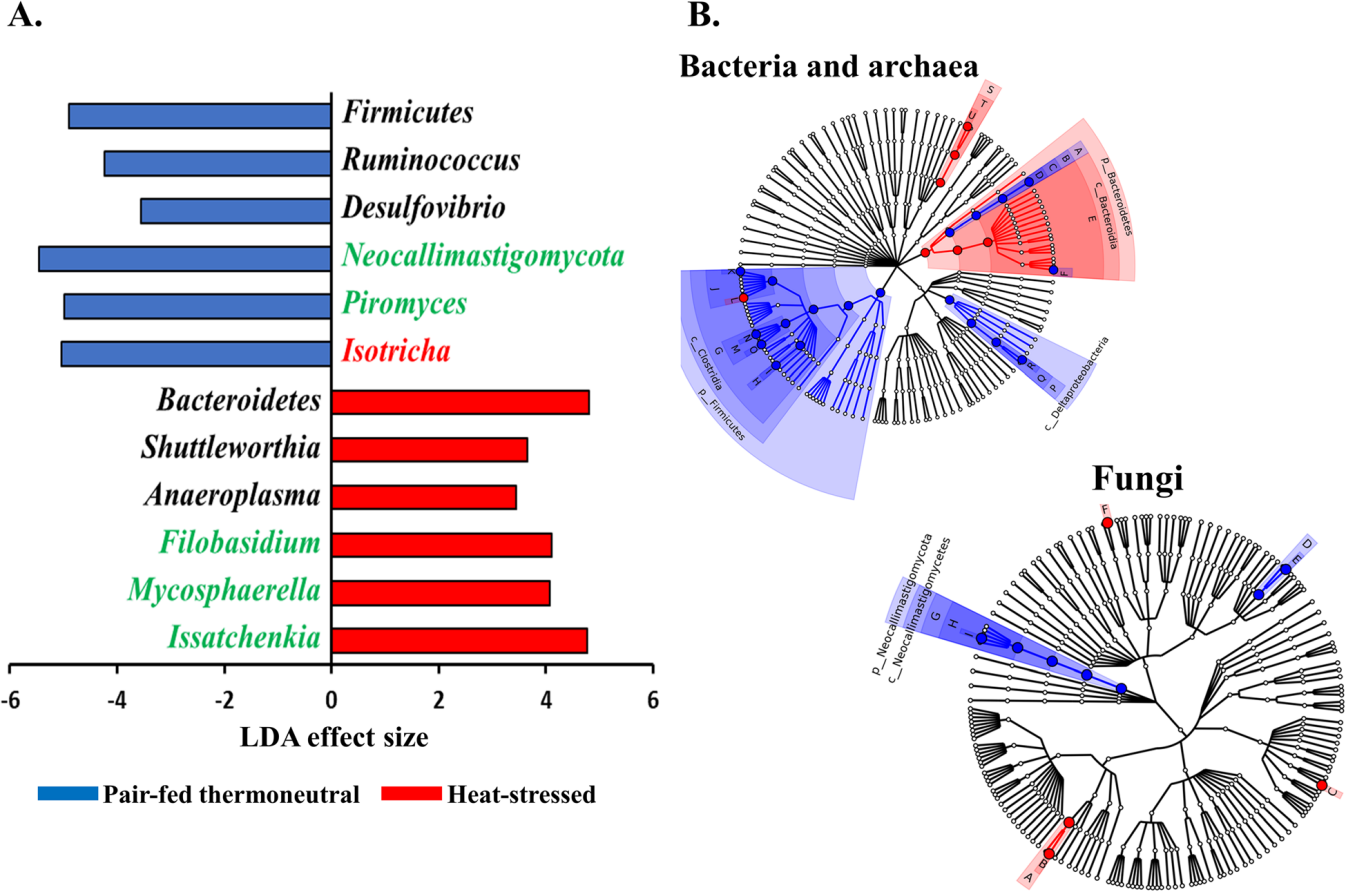
Differentially abundant rumen microbial taxa between heat-stressed (HS) cows and the pair-fed thermal neutral (PFTN) control cows, which were detected using LEfSe with an LDA effect size ≥ 2. Only classified prevalent taxa (each detected in at least 50% of the samples) were visualized with additional taxonomic lineages of prokaryotes (**B**) and fungi (**C**) embedded in a respective cladogram

**Fig. 5:**
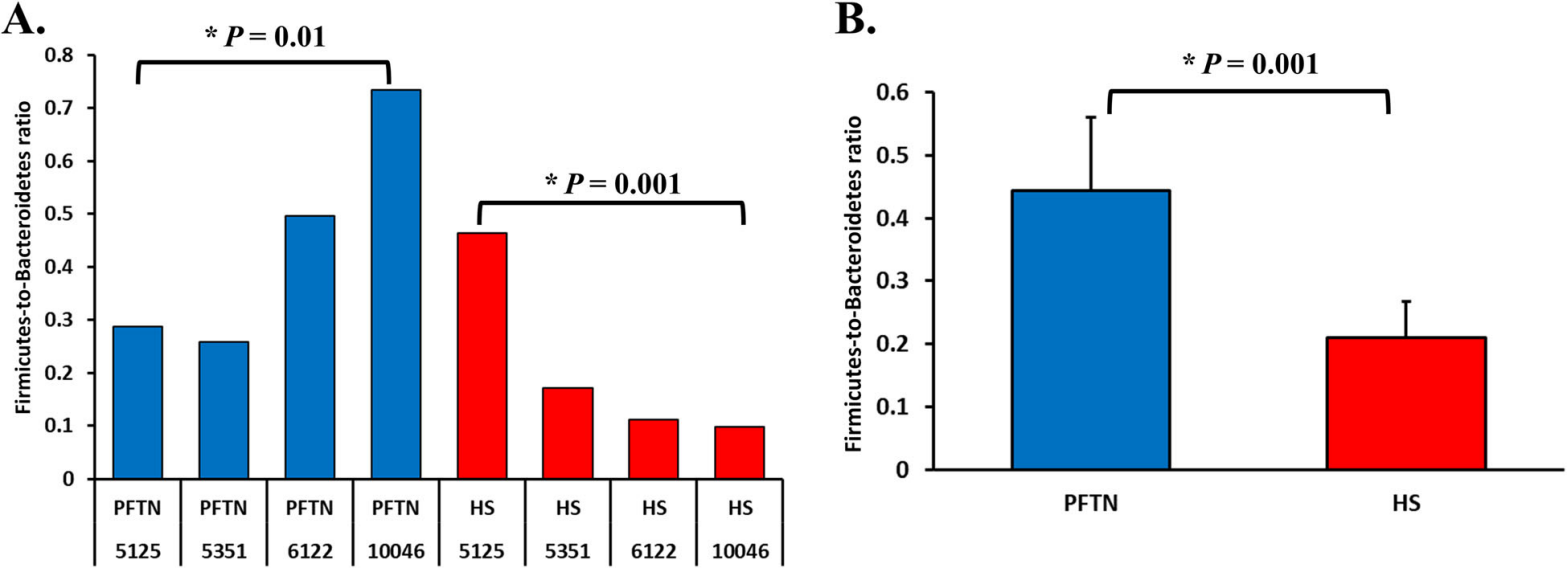
Firmicutes-to-Bacteroidetes ratio (**A**) among the four pair-fed thermal neutral (PFTN) control cows or the heat-stressed (HS) cows and (**B**) between the two groups of cows. * indicates *P* < 0.05. *P*-value: treatment: 0.001; period: 0.002; T × P: 0.198

Among the taxonomically classified genera, 24 genera in 13 phyla of prokaryotes, 29 genera in three phyla of fungi, and seven genera in the same subclass (Trichostomatia) of protozoa were found in at least 50% of all the rumen samples (Additional file 2: Fig. S1), and they were considered as prevalent taxa. Analysis using LEfSe identified differentially abundant microbial phyla and genera between PFTN control and HS cows (Fig. 4). At the phylum level, Firmicutes and Neocallimastigomycota were enriched in PFTN control cows, while Bacteroidetes was enriched in the HS cows. This resulted in a decreased (*P* < 0.001) Firmicutes-to-Bacteroidetes ratio in the HS cows (Fig. 5). There was also a period (before and after the animals were crossed over to the other thermal condition) effect on the Firmicutes-to-Bacteroidetes ratio (*P* = 0.002) though there was no thermal condition by period interaction with respect to this ratio (*P* = 0.20). Two of the cows (cows 5125 and 10,046) also exhibited a significant difference (*P* < 0.05) in Firmicutes-to-Bacteroidetes ratio under either thermal condition (Fig. 5). Different from the other cows, cow 5125 did not decrease the Firmicutes-to-Bacteroidetes ratio when heat stressed. Among the taxonomically classified genera of bacteria, *Ruminococcus* and *Desulfovibrio* were enriched in the PFTN control cows, while *Shuttleworthia* and *Anaeroplasma* expanded their relative abundance in the HS cows. Another six unclassified genera (one each assigned to *Clostridiales*, WCHB1–25, *Lachnospiraceae*, *Ruminococcaceae*, Bacteroidetes, and *Paraprevotellaceae*) were also enriched in the PFTN control cows (data not shown). Although not identified as a biomarker by the LEfSe analysis, *Prevotella* tended to increase (*P* < 0.093) its relative abundance in the HS cows, by more than 10% (17.1% vs. 27.9%). Of the prevalent fungal genera, *Filobasidium*, *Mycosphaerella*, and *Issatchenkia* were enriched in the HS cows, while *Piromyces* was enriched in the PFTN control cows (Fig. 4). Of the protozoa, the genus *Isotricha* was enriched in the PFTN control cows, and the HS did not increase the relative abundance of any of the protozoal genera.

### Heat stress affects some predicted functions of prokaryotes

**Fig. 6:**
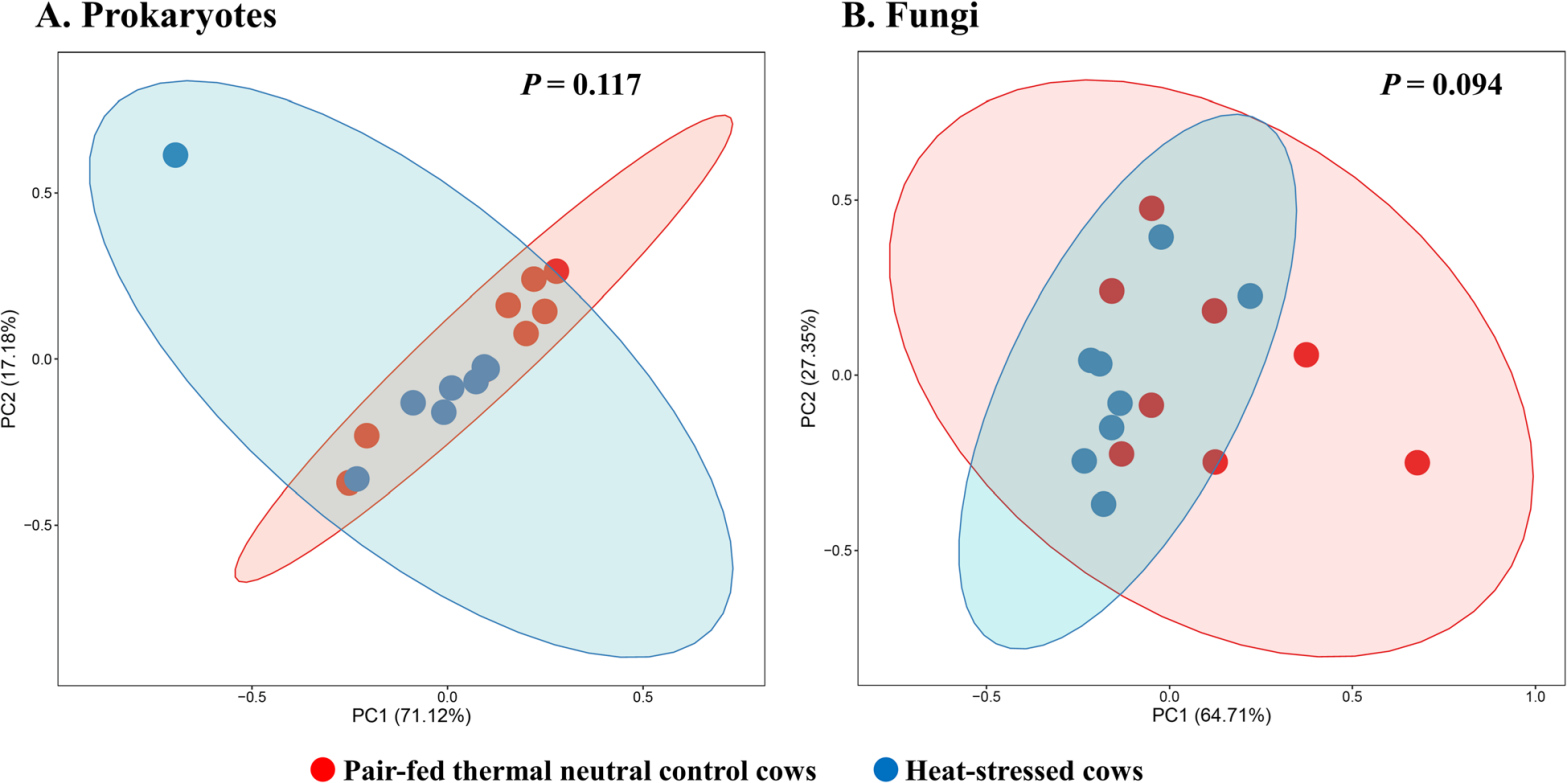
PCA plots (based on Bray-Curtis dissimilarity) comparing the overall functional features of the rumen microbiota of prokaryotes (**A**) and fungi (**B**). Functional features were predicted using PICRUSt2 and the ExPASy ENZYME database. *P*-values were based on PERMANOVA (9,999 permutations)

The overall numbers of functional features predicted using the 16S rRNA gene and PICRUSt2 with six different reference databases were not changed by HS (Additional file 1: Table S2). Based on the relative abundances of predicted features (indicated by EC numbers), the overall distributions of the prokaryotic enzyme profiles were not affected by HS (*P* = 0.117), while the fungal enzyme profiles tended (*P* = 0.094) to be affected (Fig. 6).

Comparative analysis using LEfSe identified the biomarker enzymes (indicated by EC numbers) that were differentially abundant (*P* < 0.05, LDA score > 2) between the HS cows and PFTN control cows, particularly among the major enzymes each with a relative abundance > 0.1% for at least one of the thermal conditions (Table 2 and Table 3). Overall, 18 and 9 EC numbers of prokaryotes were enriched and reduced, respectively by HS. These included three oxidoreductases (EC 1.-.-.-), four transferases (EC 2.-.-.-) including three transferases transferring phosphorus-containing groups (EC 2.7.-.-), seven hydrolases (EC 3.-.-.-) including three hydrolases acting on ester bonds (EC 3.1.-.-) and three hydrolases acting on peptide bonds (peptidases, EC 3.4.-.-), and two isomerases (EC 5.-.-.-). Of the fungal enzymes, six and 14 were enriched and reduced, respectively by HS (Table 2 and Table 3). These included three oxidoreductases (EC 1.-.-.-) including two oxidoreductases acting on the CH-OH group of donors.

**Table 2:** Predicted prokaryotic and fungal enzymes^*^ significantly (*P* < 0.05) enriched (as detected using LEfSe) in the ruminal microbiota of heat-stressed (HS) cows relative to pair-fed thermal neutral (PFTN) control cows

Enzymes	EC number	Relative abundance, %		SEM	LDA score	***P***-value
		PFTN	HS			
Predicted enzymes from prokaryotic 16S rRNA genes						
Malate dehydrogenase	EC:1.1.1.37	0.119	0.140	0.007	2.036	0.046
Succinate dehydrogenase (quinone)	EC:1.3.5.1	0.248	0.276	0.010	2.142	0.046
Glycine dehydrogenase (aminomethyl-transferring)	EC:1.4.4.2	0.172	0.210	0.010	2.248	0.046
Thiamine-phosphate diphosphorylase	EC:2.5.1.3	0.186	0.205	0.006	2.026	0.046
Fructokinase	EC:2.7.1.4	0.137	0.159	0.005	2.005	0.036
Galactokinase	EC:2.7.1.6	0.149	0.172	0.006	2.068	0.016
Aspartate kinase	EC:2.7.2.4	0.282	0.311	0.007	2.126	0.036
Type I site-specific deoxyribonuclease	EC:3.1.21.3	0.416	0.455	0.011	2.314	0.046
Ribonuclease Z	EC:3.1.26.11	0.130	0.150	0.006	2.013	0.046
Phosphoribosyl 1,2-cyclic phosphate phosphodiesterase	EC:3.1.4.55	0.106	0.133	0.008	2.105	0.036
Tripeptide aminopeptidase	EC:3.4.11.4	0.128	0.151	0.006	2.055	0.021
Peptidyl-dipeptidase Dcp	EC:3.4.15.5	0.185	0.222	0.011	2.248	0.046
C-terminal processing peptidase	EC:3.4.21.102	0.376	0.426	0.016	2.401	0.036
XTP/dITP diphosphatase	EC:3.6.1.66	0.271	0.299	0.010	2.173	0.046
N-acetylmuramic acid 6-phosphate etherase	EC:4.2.1.126	0.080	0.110	0.007	2.154	0.036
Peptidylprolyl isomerase	EC:5.2.1.8	1.012	1.140	0.039	2.770	0.046
Mannose-6-phosphate isomerase	EC:5.3.1.8	0.155	0.181	0.008	2.125	0.027
Carbamoyl-phosphate synthase (glutamine-hydrolyzing)	EC:6.3.5.5	0.364	0.398	0.008	2.239	0.016
Predicted fungal enzymes from fungal ITS1						
Pyridoxine 4-dehydrogenase	EC:1.1.1.65	0.196	0.327	0.035	2.784	0.021
Aryl-alcohol dehydrogenase (NADP^(+)^)	EC:1.1.1.91	0.104	0.129	0.006	2.022	0.036
Unspecific monooxygenase	EC:1.14.14.1	0.621	1.225	0.153	3.429	0.021
D-aspartate oxidase	EC:1.4.3.1	0.080	0.104	0.006	2.030	0.027
Glucuronosyltransferase	EC:2.4.1.17	0.048	0.160	0.032	2.724	0.046
Phospholipase D	EC:3.1.4.4	0.181	0.267	0.024	2.610	0.036

^*^ Only the major predicted enzymes with a relative abundance > 0.1% for at least one of the environmental conditions were presented

**Table 3:** Predicted prokaryotic and fungal enzymes^*^ significantly (*P* < 0.05) reduced (as detected using LEfSe) in the ruminal microbiota of heat-stressed (HS) cows relative to pair-fed thermal neutral (PFTN) control cows

Enzymes	EC number	Relative abundance, %		SEM	LDA score	***P***-value
		PFTN	HS			
Predicted enzymes from 16S rRNA genes						
Glutamate-5-semialdehyde dehydrogenase	EC:1.2.1.41	0.100	0.077	0.005	2.056	0.021
Hydroxylamine reductase	EC:1.7.99.1	0.108	0.085	0.005	2.013	0.012
3-deoxy-7-phosphoheptulonate synthase	EC:2.5.1.54	0.105	0.065	0.011	2.273	0.036
Non-specific serine/threonine protein kinase	EC:2.7.11.1	0.280	0.244	0.011	2.199	0.046
Glutamate 5-kinase	EC:2.7.2.11	0.100	0.077	0.005	2.055	0.021
Serine-type D-Ala-D-Ala carboxypeptidase	EC:3.4.16.4	0.276	0.240	0.013	2.213	0.036
Oxaloacetate decarboxylase	EC:4.1.1.3	0.175	0.134	0.009	2.327	0.021
Asparaginyl-tRNA synthase (glutamine-hydrolyzing)	EC:6.3.5.6	0.156	0.103	0.019	2.432	0.036
Glutaminyl-tRNA synthase (glutamine-hydrolyzing)	EC:6.3.5.7	0.190	0.132	0.018	2.438	0.036
Predicted enzymes from fungal ITS1						
Superoxide dismutase	EC:1.15.1.1	0.356	0.321	0.007	2.190	0.012
Cytochrome-b5 reductase	EC:1.6.2.2	0.274	0.237	0.009	2.233	0.027
Cytochrome-c oxidase	EC:1.9.3.1	0.483	0.446	0.009	2.181	0.036
Histone-lysine N-methyltransferase	EC:2.1.1.43	0.293	0.261	0.007	2.160	0.016
Phosphatidylinositol N-acetylglucosaminyltransferase	EC:2.4.1.198	0.261	0.224	0.009	2.227	0.027
Hexokinase	EC:2.7.1.1	0.156	0.132	0.005	2.049	0.006
6-phosphofructo-2-kinase	EC:2.7.1.105	0.118	0.095	0.006	2.007	0.046
Ribose-phosphate diphosphokinase	EC:2.7.6.1	0.249	0.219	0.007	2.106	0.021
Mannose-1-phosphate guanylyltransferase	EC:2.7.7.13	0.217	0.188	0.008	2.128	0.036
Ethanolaminephosphotransferase	EC:2.7.8.1	0.132	0.109	0.006	2.046	0.021
Triacylglycerol lipase	EC:3.1.1.3	0.493	0.444	0.012	2.342	0.036
Transferred entry: 4.6.1.16	EC:3.1.27.9	0.206	0.181	0.006	2.038	0.016
Alkaline phosphatase	EC:3.1.3.1	0.138	0.114	0.005	2.027	0.012
Glutamate decarboxylase	EC:4.1.1.15	0.147	0.125	0.007	2.022	0.046

Only the major predicted enzymes with a relative abundance > 0.1% for at least one of the environmental conditions were presented * is superscript

## Discussion

Although not affecting ruminal temperature [54], heat stress has been shown to alter the ruminal microbiota in dairy cows [25, 29, 30]. However, all the studies in the literature did not eliminate the negative effect on feed intake [17, 55], failing to separate the direct effect from the indirect effect caused by decreased feed intake. In this study, we eliminated that confounding factor using pair-feeding so that we could investigate how heat stress can affect the ruminal microbiota independent of its effect on feed intake. We used a small number of animals (*n* = 4 per thermal conditions) because it is prohibitive to use many animals in large environmental chambers, which are costly to purchase and operate. We used a 2 × 2 crossover design to minimize the animal effect. Overall, the heat stress conditions (THI = 87.2 during daytime and THI = 81.8 during nighttime) affected the three kingdoms (prokaryotes, fungi, and protozoa) differently. Except for the genus richness of protozoal microbiota, the heat stress treatment did not affect any of the α-diversity metrics of any of the three kingdoms. This is in general agreement with the results (only bacterial microbiota was analyzed) of another study on Holstein cows, except for Chao1 richness estimates [25] and Hanwoo steers [30]. These results suggest that a decrease in feed intake may not exacerbate the effect of heat stress on the α-diversity of the ruminal microbiota.

The heat stress treatment significantly altered the protozoal microbiota as shown by PCoA and PERMONAVA analyses and tended to decrease Shannon’s index and evenness. Analysis using LEfSe revealed a significant decrease in the relative abundance of *Isotricha*. The relative abundance of *Dasytricha* was 3.92-fold lower in HS cows compared to that of PFTN cows. Heat stress is accompanied by low rumen pH due to decreased saliva secretion [3], and indeed, the pH of the rumen was lower in the HS cows than in the PFTN control cows (pH 6.7 vs. 6.3, *P* = 0.01) [11]. The lower rumen pH in the HS cows than the PFTN control cows might be a major reason explaining the observed effect of heat stress on rumen protozoal microbiota, which are inhibited by low ruminal pH [56]. The decrease of *Isotricha* and *Dasytricha* also agrees with the report that these two genera of holotrichs are probably more sensitive to ruminal pH than other protozoal genera [57]. In the present study, we did not determine if the heat stress treatment decreased total rumen protozoal counts. No study in the literature has included protozoa in the evaluation of the effect of heat stress on ruminal microbiota. Given the sensitivity to rumen pH, rumen protozoa probably could lose abundance in heat stressed cows, but quantitative analyses using microscopic counting or qPCR are needed to verify this surmise in future studies. It should be noted that there was a significant period effect with respect to the impact of heat stress on the protozoal Shannon’s index, Simpson’s index, and evenness. It remains to be determined how the residual effect of period (thermal vs. heat stress in a crossover design) can affect the above α-diversity metrics. Such period effect should be taken into consideration in the design of future studies.

Rumen fungi are recognized for their fibrolytic activity [58, 59], and many of the gut fungi have not been classified [60]. Although all the taxonomically known genera of rumen fungi were detected in the PFTN control cows, in the present study, heat stress did not affect the diversity (α or β) of the fungal microbiota. However, some genus-level taxa disappeared while others appeared in the HS cows. The heat stress also considerably enriched the genera *Mycosphaerella*, *Filobasidium*, and *Issatchenkia*, while diminishing Neocallimastigomycota and the genus *Piromyces*. Except for *Piromyces,* all those genera were not commonly found in the rumen and had low relative abundance. The animal study [11] showed the digestibility of all the nutrients (NDF, DM, ADF, CP, EE, and OM) were significantly higher (*P* < 0.05, without significant period effect) in the HS cows than in the PFTN control cows even though heat stress impaired lactation performance [decreasing milk yield by 17.0% (25.9 vs. 21.5 kg/d), milk protein by 4.1% (2.68% vs. 2.57% of milk), milk protein yield by 19% (705 vs. 571 g/d), 4% fat-corrected milk by 23% (28.0 vs. 21.6 kg/d), and fat yield by 19% (1096 vs. 884 g/d)]. The authors attributed the improvements of feed digestion to decreased rumen motility and thus passage rate [11], both of which are common in HS cows [61]. Heat stress also tended to alter the predicted fungal features and decreased many of the fungal enzymes, especially hydrolases including phosphotransferase and esterases. This discrepancy is consistent with the overall minor role of rumen fungi in feed digestion. To the best of our knowledge, no study in the literature has examined how heat stress could affect the rumen fungal microbiota. Future research is needed to quantitatively determine to what extent heat stress affects the rumen fungal populations and their function.

Prokaryotes, which account for the majority of the ruminal microbiota, were analyzed in all studies on ruminal microbiota, but ruminal fungi and protozoa were rarely analyzed. In the present study, heat stress altered the overall prokaryotic microbiota (significantly if based on unweighted UniFrac analysis, data not shown). This finding is consistent with the report of Kim et al. [26] but does not corroborate the report by Zhao et al. [25]. However, the three studies differ in the actual THI imposed on the cows, making comparison and interpretation of the results difficult. The heat stress conditions of the present study significantly increased the relative abundance of Bacteroidetes (primarily Gram-negative bacteria), while decreasing that of Firmicutes (primarily Gram-positive bacteria). Such a phylum-level shift was reported in the ruminal microbiota in heat-stressed goats [29] and the fecal microbiota of heat-stressed lactating dairy cows [62]. Future research is needed to verify if this Firmicutes to Bacteroidetes shift has anything to do with their Gram staining features and if the Firmicutes-to-Bacteroidetes ratio of the rumen or fecal microbiota can serve as a microbial biomarker of heat stress. In the recent studies, a shift of other bacterial phyla was reported, including a decrease in Fibrobacteres but an increase in Fusobacteria, Tenericutes, and Cyanobacteria in heat-stressed lactating Holstein cows [26] and a decrease in Proteobacteria and Chloroflexi in heat-stressed Hanwoo steers [30]. In the present study, these phyla were not affected by heat stress. The discrepancy might be attributable to the decreased feed intake by the HS cows in the two cited studies.

Many bacterial genera (including some that have no cultured species) were also detected exclusively under each thermal condition. Similar to the case of fungi, nearly all these genera had a very low relative abundance. Given the very high Good’s coverage (≥ 99.9%), those exclusively detected genera were probably very minor ones selected by each thermal condition. Only three taxonomically known genera (unclassified genus-level taxa were not discussed or compared with other studies because it is not possible) were enriched (*Prevotella, Shuttleworthia,* and *Anaeroplasma*) or diminished (*Ruminococcus* and *Desulfovibrio*) by heat stress. The observed decrease in *Ruminococcus* in the HS cows contradicts the report for heat-stressed goats [29] but concurs with the reports for heat-stressed dairy cows [25, 26] or Hanwoo steers [30]. On the other hand, none of the genera (except for *Prevotella*) detected in the HS cows in other recent studies was detected in the present study (Additional file 1: Table S3). Among other factors, the elimination of the confounding effect on feed intake might be attributable to these discrepancies. The “heat-stress resistant” microbes that were enriched or exclusively found in the rumen of heat-stressed cows may be useful as potential probiotics for cows under heat stress.

The decrease in *Ruminococcus*, which is a highly fibrolytic genus [63], and *Desulfovibrio*, which is a sulfate-reducing VFA-utilizing genus [64], in the HS cows, seems contrary to the increased fiber digestibility (by 21.4%, dry matter) and VFA concentration (by 82.9%, total VFA) in those HS cows. The increase in *Shuttleworthia*, which is saccharolytic producing acetic, butyric, and lactic acids as major fermentation products [65], and *Anaeroplasma*¸ which ferments sugars to primarily acetic, formic, propionic, lactic, and succinic acids [66], is consistent with the increased rumen VFA concentration in the HS cows, but their absolute abundance needs to be quantified to determine if and to what extent they can contribute to the increased VFA in the HS cows. As the largest genus, *Prevotella* increased its relative abundance by > 10% (27.9% vs. 17.1%) in the HS cows, and the large increase of this versatile genus might explain some of the observed changes in nutrient digestibility and fermentation characteristics [11]. It should be noted that many taxonomically unknown genus-level taxa were exclusively found in either the HS cows or the PFTN control cows, suggesting that they may adapt or be susceptible to the rumen environment created by heat stress. Their role and contribution to the rumen function in HS cows remain to be determined.

Using two different network analyses (FastSpar and CoDiNA), we identified some co-occurrence and mutual-exclusion relationships between some taxonomically known genera exclusively detected for each thermal condition. Interestingly, more co-occurrence or mutual exclusion relationships were found in the ruminal microbiota of the PFTN control cows than the HS cows (17 vs. 10). The two thermal conditions also corresponded to co-occurrence and mutual exclusion of very different genera. In the literature, only one study examined co-occurrence and mutual exclusion of rumen microbial taxa while determining the effect of heat stress on the ruminal microbiota in goats [29]. Mutual exclusion between *Ruminococcus* and *Prevotella* was reported, but it was not stated if it was in the rumen of thermal control goats or heat-stressed goats. Because co-occurrence and mutual exclusion were based on correlation of relative abundance between genera, they may not necessarily be attributable to mutualism or antagonism, respectively. However, the co-occurrence and mutual exclusion relationships detected exclusively for each thermal condition suggest that heat stress can alter the response of those genera.

All the recent studies evaluated the impact of heat stress on the taxonomic diversity and composition of the ruminal microbiota [25, 26, 29, 30], but only two studies [26, 29] evaluated the effect on the functional features of the ruminal microbiota. In the present study, we predicted the functional features based on the 16S rRNA gene sequence data using PICRUSt2. The overall functional features of prokaryotes did not differ between the HS cows and the PFTN control cows. The discrepancy between the effect of heat stress on the rumen prokaryotic microbiota (PERMANOVA *P* = 0.068) and on the predicted prokaryotic functional profiles (PERMANOVA *P* = 0.117) is consistent with the functional redundancy of the ruminal microbiota [67]. The heat stress did affect some of the predicted enzymes, particularly striking the enrichment of seven hydrolases including those acting on ester bonds and peptide bonds. This corroborates the increased nutrient digestibility reported in the HS cows [11]. The increased retention time corresponding to the slowed passage rate might have increased some hydrolytic bacteria. However, the minimal impact of heat stress on the rumen function does not mirror the impaired lactation performance (by 17.0%) in the HS cows. The prediction of functions using phylogenetic markers certainly has limitations. In future studies, direct and quantitative functional analyses using other technologies, such as metatranscriptomics, metabolomics, and guild-specific tests, can better help determine how heat stress affects rumen functions. Nonetheless, these results suggest that heat stress may decrease lactation performance in dairy cows by largely impairing the physiology and metabolism, not the rumen function. Indeed, heat stress was found to decrease milk protein synthesis in the mammary glands [11] and other important biological functions such as metabolism, immunity, inflammation, changes in behavior, and antioxidant capacity [8, 13, 14, 68]. A recent study using Holstein heifers showed that fermented herbal tea residues improved some physiological indices related to heat stress, including respiratory frequency, rectal temperature, concentrations of immunoglobulins, antioxidant capacity, and serum concentrations of heat stress-related parameters [69]. In finishing male pigs, *Saccharomyces cerevisiae boulardii* CNCM I-1079 supplementation was shown to help cope with heat stress by modulating their gut microbiome and feeding behavior [70]. Future studies are warranted to evaluate if these approaches can be applied to lactating dairy cows.

As global warming continues to worsen, heat stress will affect animals and humans, particularly those living in tropical regions, to a greater extent than what has been reported. In the present study, we focused on the ruminal microbiome because of its critical role in feed digestion and supplying much of the nutrients to cows. Other studies have shown that heat stress could also affect the fecal microbiome [71–73], a proxy of the microbiome of large intestines. Although not reported, heat stress can also affect the gut microbiome of animals and humans. Pair-feeding should be used in future studies to examine the effect of heat stress on the gut microbiome thereof.

## Conclusions

Heat stress affected the ruminal microbiota structure of the three kingdoms (prokaryotes, fungi, and protozoa) to a different extent in the absence of the confounding factor of feed intake. Heat stress selected some taxa of bacteria, fungi, and protozoa while decreasing others. Heat stress also altered the co-occurrence and mutual exclusion between some genera. Firmicutes-to-Bacteroidetes ratio decreased in heat-stressed cows, and several genera decreased (*Ruminococcus*, *Desulfovibrio*, *Piromyces*, *Isotricha*) or increased (*Anaeroplasma*, *Shuttleworthia*, *Filobasidium*). The heat stress conditions had little impact on the predicted functions of the rumen prokaryotic or fungal microbiota. The effect of heat stress on the ruminal microbiota observed in the present study should be primarily attributed to its adverse effect on the host metabolism, physiology, and behavior. Some of the “heat-stress resistant” bacteria and fungi may be useful as potential probiotics for cows under heat stress.

## Supplementary Information

**Additional file 1: Table S1.** DADA2 denoising statistics of the ruminal microbiota of the heat-stressed (HS) cows and the pair-fed thermal neutral (PFTN) control cows. **Table S2.** Counts of PICTRUSt2-predicted functional features in the ruminal microbiota of heat-stressed (HS) cows and pair-fed thermal neutral (PFTN) control cows. **Table S3.** Comparison of the taxonomically known genera of bacteria affected by heat stress.

**Additional file 2: Fig. S1.** Relative abundance of prevalent (found in at least 50% of all the samples) classified prokaryotic phyla (**A**), prokaryotic genera (**B**), fungal phyla (**C**), fungal genera (**D**), and protozoal genera (**E**).

## Data Availability

The 16S rRNA gene amplicon sequences, ITS1 amplicon sequences, and 18S rRNA gene sequences generated for this study can be found in the NCBI Sequence Read Archive (SRA) under BioProject PRJNA562317.

## Abbreviations

ASVs: Amplicon sequence variants
EC: Enzyme Commission
GHG: Greenhouse gases
HS: Heat stress; Heat-stressed
IMG: Integrated Microbial Genomes
LEfSe: Linear discriminant analysis effect size
PFTN: Pair-fed thermoneutrality; Pair-fed thermal neutral
PCA: Principal components analysis
PCoA: Principal coordinates analysis
PERMANOVA: Permutational multivariate analysis of variance
THI: Temperature-humidity index
