# Supplementary material for: Heat stress impacts the multi-domain ruminal microbiota and some of the functional features independent of its effect on feed intake in lactating dairy cows
Source: J Anim Sci Biotechnol. 2022 Jun 15;13:71. doi: 10.1186/s40104-022-00717-z (PMC9199214; doi:10.1186/s40104-022-00717-z)
Supplement: Supplementary file 1 — Additional file 1: Table S1. DADA2 denoising statistics of the ruminal microbiota of the heat-stressed (HS) cows and the pair-fed thermal neutral (PFTN) control cows. Table S2. Counts of PICTRUSt2-predicted functional features in the ruminal microbiota of heat-stressed (HS) cows and pair-fed thermal neutral (PFTN) control cows. Table S3. Comparison of the taxonomically known genera of bacteria affected by heat stress. [file 40104_2022_717_MOESM1_ESM.docx]

| **Table S1.** DADA2 denoising statistics of the ruminal microbiota of the heat-stressed (HS) cows and the pair-fed thermal neutral (PFTN) control cows. | | | | | | | | | | | |
| --- | --- | --- | --- | --- | --- | --- | --- | --- | --- | --- | --- |
|  | **Bacteria & methanogens** | | |  | **Fungi** | | |  | **Protozoa** | | |
|  | Treatments | | SEM |  | Treatments | | SEM |  | Treatments | | SEM |
|  | PFTN | HS |  |  | PFTN | HS |  |  | PFTN | HS |  |
| No. of input paired reads | 15,953 | 15,770 | 713 |  | 12,712 | 18,122 | 1,500 |  | 24,348 | 26,555 | 1709 |
| Quality-filtered sequences | 14,053 | 14,210 | 703 |  | 12,696 | 18,072 | 1,492 |  | 22,574 | 24,824 | 1594 |
| Denoised sequences | 13,006 | 13,210 | 671 |  | 12,653 | 18,052 | 1,494 |  | 22,522 | 24,783 | 1593 |
| Chimera-filtered sequences | 12,194 | 12,314 | 625 |  | 12,562 | 17,979 | 1,502 |  | 21,856 | 23,221 | 1561 |
| Taxa-filtered sequences^*^ | 12,190 | 12,277 | 621 |  | 12,562 | 17,979 | 1,502 |  | 21,856 | 23,221 | 1561 |

^*^After filtering out the sequences of mitochondria and chloroplast and the sequences unassignable to any taxa.

| **Table S2.** Counts of PICTRUSt2-predicted functional features in the ruminal microbiota of heat-stressed (HS) cows and pair-fed thermal neutral (PFTN) control cows. | | | | | | |
| --- | --- | --- | --- | --- | --- | --- |
| Databases | Treatments (Trt^*^) | | SEM | *P*-values | | |
|  | PFTN^*^ | HS^*^ |  | Trt | Period (P) | T×P |
| KEGG orthologs | 4,382 | 4,142 | 182.24 | 0.522 | 0.193 | 0.416 |
| KEGG pathways | 129 | 126 | 1.29 | 0.279 | 0.418 | 0.241 |
| KEGG modules | 248 | 241 | 4.83 | 0.488 | 0.170 | 0.310 |
| COG | 3,593 | 3,430 | 98.91 | 0.410 | 0.153 | 0.309 |
| EC | 1,459 | 1,390 | 39.9 | 0.386 | 0.114 | 0.429 |
| Fungal-EC | 942 | 946 | 15.14 | 0.913 | 0.554 | 0.214 |
| PFAM | 5,257 | 5,020 | 166.66 | 0.480 | 0.143 | 0.403 |
| MetaCyc pathways | 299 | 286 | 7.83 | 0.412 | 0.131 | 0.525 |

^*^Thermal treatment (heat stress vs. pair-fed thermoneutrality)

| **Table S3.** Comparison of the taxonomically known genera^*^ of bacteria affected by heat stress | | | | |
| --- | --- | --- | --- | --- |
| Animal | THI (TN / HS)^**^ | Genera increased by heat stress | Genera decreased by heat stress | References |
| Jersey cows^#^ | 69.9 / 87.5 | *Staphylococcus*  *Clostridium* | *Streptomyces*  *Pseudomonas* | [1] |
| Holstein cows^#^ | 71 / 87 | *Treponema*  *Ruminobacter*  *Streptococcus* | *Acetobacter* | [2] |
| Hanwoo steers^#^ | 69.6 / 87.5 | *Prevotella*  *Lactobacillus*  *Ruminobacter* | none | [3] |
| Holstein cows^√^ | 65.5 / 81.8 – 87.2 | *Prevotella*  *Anaeroplasma*  *Shuttleworthia* | *Ruminococcus*  *Desulfovibrio* | This study |

^*^Some of the studies reported classes, orders, and families that were affected by heat stress, but they were not shown.

^**^THI: thermal humidity index; TN: thermal neutral; HS: heat stress.

^#^ad libitum fed.

^√^pair-fed.

**References**

1. Kim D-H, Kim M-H, Kim S-B, Son J-K, Lee J-H, Joo S-S, et al. Differential dynamics of the ruminal microbiome of Jersey cows in a heat stress environment. Animals. 2020;10:1127. <https://doi.org/10.3390/ani10071127>.

2. Zhao S, Min L, Zheng N, Wang J. Effect of heat stress on bacterial composition and metabolism in the rumen of lactating dairy cows. Animals. 2019;9:925. <https://doi.org/10.3390/ani9110925>.

3. Baek YC, Choi H, Jeong J-Y, Lee SD, Kim MJ, Lee S, et al. The impact of short-term acute heat stress on the rumen microbiome of Hanwoo steers. J Anim Sci Technol. 2020;62:208-17. <https://doi.org/10.5187/jast.2020.62.2.208>.
