# Supplementary figures and images for: Heat stress impacts the multi-domain ruminal microbiota and some of the functional features independent of its effect on feed intake in lactating dairy cows
Source: J Anim Sci Biotechnol. 2022 Jun 15;13:71. doi: 10.1186/s40104-022-00717-z (PMC9199214; doi:10.1186/s40104-022-00717-z)

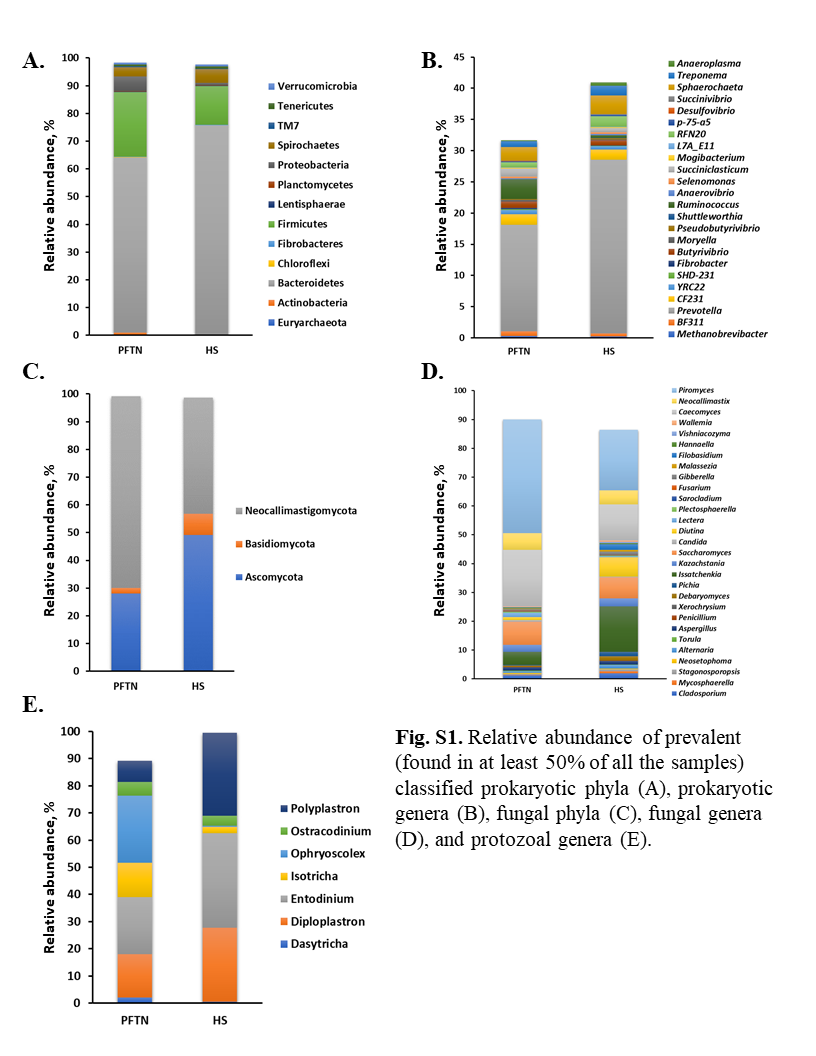

Supplement: Supplementary file 2 — Additional file 2: Fig. S1. Relative abundance of prevalent (found in at least 50% of all the samples) classified prokaryotic phyla (A), prokaryotic genera (B), fungal phyla (C), fungal genera (D), and protozoal genera (E). [file 40104_2022_717_MOESM2_ESM.tif]
